# Serum α_1_-Proteinase Inhibitor, Calprotectin, and S100A12 Concentrations in the Characterization of Pancreatitis in Dogs

**DOI:** 10.3390/vetsci10070428

**Published:** 2023-07-01

**Authors:** Annina N. Jandel, Romy M. Heilmann, Henri Sander, Jörg M. Steiner, Niels Grützner, Panagiotis G. Xenoulis

**Affiliations:** 1 Department for Small Animals, College of Veterinary Medicine, University of Leipzig, DE-04103 Leipzig, Germany; anninajandel@gmail.com (A.N.J.); h.sander710@gmail.com (H.S.); 2 Gastrointestinal Laboratory, School of Veterinary Medicine and Biomedical Sciences, Texas A&M University, College Station, TX 77843, USA; jsteiner@cvm.tamu.edu (J.M.S.); pxenoulis@gmail.com (P.G.X.); 3 Ruminant and Swine Clinic, Free University of Berlin, DE-14165 Berlin, Germany; degruetz@web.de; 4 VetaRegio GmbH, DE-06366 Köthen, Germany; 5 Clinic of Medicine, Faculty of Veterinary Science, University of Thessaly, 43100 Karditsa, Greece

**Keywords:** dog, Miniature Schnauzer, pancreatitis, serum α_1_ proteinase inhibitor, calprotectin, S100A12

## Abstract

**Simple Summary:**

Pancreatitis in dogs is an inflammatory condition that can be acute or chronic and often leads to non-specific clinical signs, including nausea, abdominal pain, and diarrhea. The Miniature Schnauzer is a breed with a predisposition to both pancreatitis and familial hypertriglyceridemia, and both conditions can affect each other. Successful treatment depends on reliable diagnosis as well as assessment of disease severity and prognosis, which are inherently difficult to assess upon initial presentation of the dog. Within this study, serum alpha_1_-proteinase inhibitor (α_1_PI; a marker of the systemic response to circulating proteases, which presumably have been released from the pancreas) and serum calprotectin and S100A12 (markers of systemic inflammation presumed to reflect an exaggerated local inflammatory response in the pancreas) were measured in 35 Miniature Schnauzers diagnosed with either a typical or an atypical presentation of pancreatitis assumed to reflect varying clinical severities. All serum markers were increased in dogs with pancreatitis, with serum α_1_PI accurately separating typically from atypically presenting dogs and a similar trend for the ratio of serum concentrations of calprotectin-to-S100A12. Thus, these markers might aid in staging disease severity in dogs with pancreatitis.

**Abstract:**

Miniature Schnauzers are predisposed to develop pancreatitis, with familial hypertriglyceridemia (HTG) described as a potential risk factor. Diagnosing pancreatitis in dogs is based on the integration of serum canine-specific pancreatic lipase (cPLI) concentration, clinical presentation, and diagnostic imaging findings. However, markers of systemic inflammation and antiprotease activity have not been extensively investigated in the characterization and prognostication of pancreatitis in dogs. Serum concentrations of alpha_1_-proteinase inhibitor (α_1_PI; as a marker of systemic antiprotease response) and calprotectin and S100A12 (as markers of systemic inflammation) were measured in serum samples from 35 Miniature Schnauzers diagnosed with pancreatitis (serum cPLI concentration >400 μg/L, clinical signs, abdominal imaging findings). These markers were evaluated for possible associations with patient characteristics, clinical presentation, risk factors for pancreatitis, and outcome. The study showed that biomarkers of systemic inflammation and antiprotease activity are commonly increased in Miniature Schnauzers with pancreatitis. Whereas serum calprotectin and S100A12 concentrations were found to have limited utility in differentiating pancreatitis presentations, serum α_1_PI concentrations and potentially also the serum calprotectin-to-S100A12 ratio might be non-invasive surrogate markers of disease severity in dogs with pancreatitis.

## 1. Introduction

Pancreatitis is a common condition in dogs, and in addition to a localized inflammatory response, it has been shown to be associated with a systemic release of inflammatory mediators, vasoactive substances, and protease inhibitors [1]. Traditionally, the gold standard for diagnosing pancreatitis in dogs is the histopathologic examination of a pancreatic tissue biopsy [2,3]. However, this method is rarely used in routine veterinary practice due to its invasiveness and associated high risk to the patient. In addition, several limitations are known to be associated with histopathology as a gold standard in the diagnosis of pancreatitis [3]. Measurement of the canine specific pancreatic lipase concentration (cPLI) in serum currently constitutes the most practical, widespread, and reliable method to aid in diagnosing canine pancreatitis [4,5], with a sensitivity of 72–94% and a specificity of 80–100% across all severities and categories of pancreatitis [6]. The result of this test is interpreted in light of the patient’s clinical signs (e.g., abdominal pain, vomiting, and nausea) and diagnostic imaging findings. Abdominal ultrasonography in dogs with pancreatitis can reveal pancreatic swelling, hypoechogenicity, and peripancreatic changes, including hyperechogenicity and free fluid accumulation with acute pancreatitis; pancreatic hyperechogenicity may also be seen in more chronic pancreatitis cases [2,3]. Contrast-enhanced ultrasound, computed tomography, and magnetic resonance imaging (MRI) are more advanced diagnostic imaging techniques to diagnose cases of pancreatitis and possible complications, such as portal vein thrombosis, but are currently rarely used in routine practice [7,8,9,10].

The acute-phase reactant C-reactive protein (CRP), which is synthesized in the liver and peaks rapidly with various inflammatory, infectious, or neoplastic conditions [11], is significantly increased in dogs with acute pancreatitis, but its correlation with disease severity has been inconsistent among different studies [12,13,14,15]. Daily monitoring of serum CRP concentrations has been shown to be predictive of prognosis, but measurement of this marker at the time of diagnosis could not predict patient outcome [16,17].

Miniature Schnauzers are reported to be predisposed to developing pancreatitis, with a 4.51-times higher prevalence of the condition compared to other breeds of dogs [18]. Primary familial hypertriglyceridemia (HTG) has been proposed as a risk factor for pancreatitis in this breed based on correlations between HTG and increased serum cPLI concentrations. In addition, Miniature Schnauzers with severe HTG (serum triglyceride concentration ≥862 mg/dL) are 4.5 times more likely to be diagnosed with pancreatitis by showing an increased serum cPLI concentration (≥200 µg/L; this study utilized the original cPLI assay [19], which was associated with a RI of < 102.1 µg/L and a cut-off value for a diagnosis of 200 µg/L) compared to Miniature Schnauzers with a normal serum triglyceride concentration [20]. In addition, Miniature Schnauzers with a history of pancreatitis are five times more likely to have hypertriglyceridemia compared to Miniature Schnauzers without such a history [21]. With increasing age, both the prevalence and severity of HTG in Miniature Schnauzers increase [22]. Previous studies discussed possible gene mutations, especially of the *SPINK1* gene, as a potential cause for primary familial HTG and the associated increased risk for pancreatitis in Miniature Schnauzers [23,24]. Secondary HTG can result from various causes, including the postprandial phase (particularly after a fatty meal), endocrine disorders (e.g., diabetes mellitus, hyperadrenocorticism), and metabolic syndrome in severely obese dogs [25], and can also increase the risk of developing or exacerbating an episode of acute pancreatitis [26,27,28].

Primary (idiopathic) HTG in Miniature Schnauzers often remains subclinical for some time, leading to a late-stage diagnosis and the risk of patients presenting with secondary conditions or complications (e.g., pancreatitis, ophthalmic signs, proteinuria, or even seizures) [25,29,30], and can be associated with a subclinical inflammatory phenotype and insulin resistance [31]. Previous studies revealed HTG to be associated with hypercalprotectinemia, a marker of systemic inflammation [32,33,34]. However, serum α_1_-proteinase inhibitor concentrations (α_1_PI, a serum antiprotease), calprotectin, and S100A12 concentrations (inflammatory markers) have not been extensively evaluated in dogs with pancreatitis.

We hypothesized that these markers of systemic inflammation and antiprotease activity are increased in Miniature Schnauzers with pancreatitis and might help to differentiate pancreatitis cases of varying severities and comorbidities in this breed. Thus, the objective of this study was to evaluate serum α_1_PI, calprotectin, and S100A12 concentrations in Miniature Schnauzers with pancreatitis and assess their potential association with patient and disease characteristics, clinical presentation and indicators of clinical disease severity, risk factors for pancreatitis, and outcome.

## 2. Materials and Methods

The patient population used for this study included 35 Miniature Schnauzers recruited at the Texas A&M University (TAMU) Gastrointestinal Laboratory. The collection of blood samples from these dogs was approved by the Clinical Research Review Committee (CRRC) at the TAMU College of Veterinary Medicine (CRRC #08-37). A standard questionnaire was used for the study to obtain a thorough patient history and information about the dogs (age, sex, and body weight; medical history, diet, medication, and prior episodes of pancreatitis; current clinical signs and outcome).

All dogs included in the study were suspected to have a current episode of pancreatitis based on an increased serum cPLI concentration (>400 µg/L), clinical signs, and abdominal imaging. Based on further diagnostic evaluation, dogs were assigned to one of the following groups: (A) cPLI consistent with pancreatitis and typical clinical presentation either with (A1) or without (A2) concurrent conditions (e.g., diabetes mellitus, neurological conditions, chronic kidney disease), or (B) cPLI consistent with pancreatitis but with an atypical clinical presentation (e.g., very mild clinical presentation and lacking some clinical signs and/or significant ultrasonographic lesions, e.g., showing only anorexia or mild depression/lethargy, or presenting with a combination of clinical signs that are suggestive of large bowel disease, such as hematochezia).

Whole blood and serum samples were obtained for a complete blood cell count, a serum biochemistry panel, including serum triglyceride concentration (reference interval [RI]: 26–108 mg/dL), and a gastrointestinal panel, including serum concentrations of cPLI, trypsin-like immunoreactivity, cobalamin, and folate. Serum concentrations of the biomarkers α_1_PI (RI: 750–1556 mg/L [35]), calprotectin (RI: 1.0–11.9 mg/L [36]), and S100A12 (RI: 33–225 µg/L [37]) were analyzed with all samples in one batch and using validated in-house immunoassays [35,36,37,38].

Statistical analyses were performed using the statistical software JMP^®^ (v.13; Cary, NC, USA) and GraphPad^®^ (v.9; San Diego, CA, USA). The normality of the data distribution was tested using a Shapiro–Wilk test, based on which the summary statistics were reported as medians (ranges) and proportions (%). Non-parametric two-group or multiple-group comparisons were performed using a Wilcoxon rank-sum test or the Kruskal–Wallis test. A Spearman correlation coefficient ρ was calculated to test for correlations between continuous variables, and likelihood ratio tests served to evaluate possible associations between categorical data. A receiver-operating characteristic (ROC) curve was constructed to calculate the sensitivity and specificity for cut-offs of continuous variables (optimal cut-offs determined by the Youden index) to distinguish groups of dogs. The statistical significance was set at *p* < 0.05 (with possible trends at *p* < 0.1). The fat content of the diet (<20 g/1000 kcal vs. ≥20 g/1000 kcal), presence of HTG (yes/no), medications considered potential risk factors for pancreatitis (yes/no), and the presence of individual clinical signs (vomiting, diarrhea, abdominal pain, anorexia, and lethargy; yes/no) were considered dichotomous variables in the analysis. In addition to univariate analysis for the effect of several parameters on the individual serum biomarker concentrations, a multivariate stepwise forward regression model using Akaike’s information criterion (AIC) was used to analyze for variables (with *p* < 0.1 in univariate analysis) that significantly affect serum concentrations of α_1_PI, calprotectin, and S100A12.

## 3. Results

Dogs included in the study had a median age of 8.9 years (range: 1.7–14.6 years), a nearly even sex distribution (52% male dogs and 48% female dogs), and a median body weight of 8.9 kg (range: 6.2–14.2 kg) (Table 1). Clinical signs (all individually recorded for 24 dogs) included vomiting (71%), lethargy (67%), abdominal pain (50%), anorexia (50%), and diarrhea (33%). Based on the available data, further stratification was possible for 35 dogs: twenty-eight of the dogs (80%) had a typical presentation of pancreatitis (group A), four of which (11%) had a concurrent condition, and the remaining seven dogs (20%) showed an atypical clinical presentation of pancreatitis (e.g., mild presentation with only anorexia, mild depression/lethargy, or mild large-bowel diarrhea).

Dietary and medication histories were available for twenty-one dogs, of which eighteen dogs (86%) received a prescription diet and two dogs (10%) a home-made diet. Complete diet information could be assessed in eighteen dogs, and two of those dogs (11%) received a low-fat commercial diet (<20 g fat/1000 kcal). Fifteen dogs (71%) received medication, and one of those dogs (7%) received a drug known to be associated with pancreatitis (phenobarbital). Seven dogs (33%; 21 available results) were diagnosed with HTG. A complete medical history was available from eighteen dogs, four of which (22%) had a previous episode of pancreatitis.

Serum α_1_PI concentrations ranged from 1011–2437 mg/L (median: 1621 mg/L), serum calprotectin from 1.2–269.7 mg/L (median: 15.5 mg/L), and serum S100A12 concentrations from 15.7–6463.5 μg/L (median: 262.3 μg/L). Increased serum α_1_PI, calprotectin, and S100A12 concentrations were detected in 19/33 dogs (58%), 20/35 dogs (57%), and 20/35 dogs (57%), respectively (Table 1). Moderate to strong correlations were detected among all three serum biomarkers, but these were neither correlated with age nor body weight (Table 2). Age, sex, body weight, number and frequency of clinical signs, and frequencies of previous pancreatitis episodes, HTG, administration of a medication that can be associated with pancreatitis, and poor outcome were not significantly different between both disease groups (Table 1).

**Figure 1:**
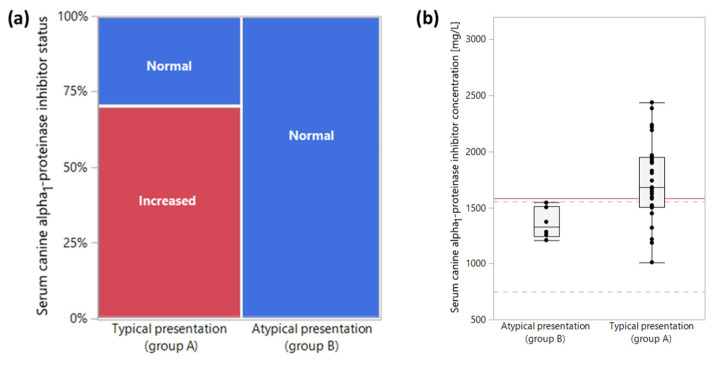

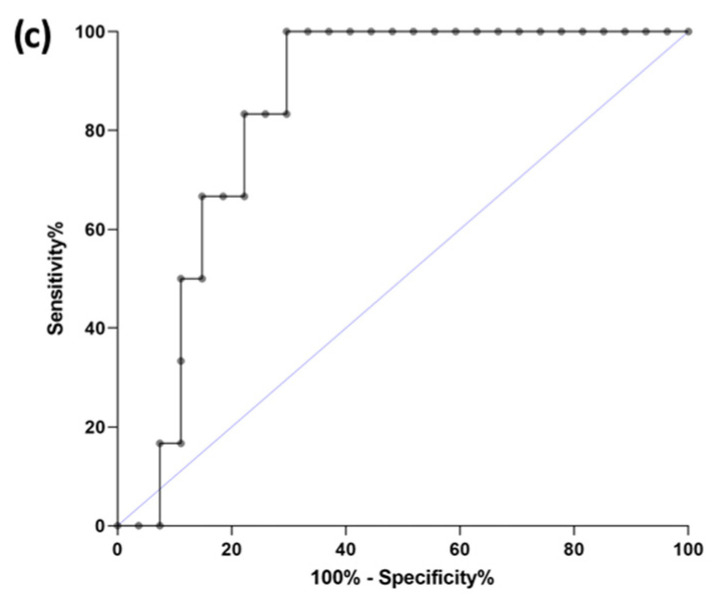
Group comparison for serum alpha_1_-proteinase inhibitor (α_1_PI) concentrations. (**a**) As opposed to the typical presentation of pancreatitis (group A), where 19 dogs (70%) had an increased serum α_1_PI concentration, this marker was not increased in any of the dogs with an atypical pancreatitis presentation (group B; *p* = 0.0005). (**b**) Dogs in group A (typical presentation of pancreatitis) had significantly higher serum α_1_PI concentrations (median: 1680 mg/L) than dogs in group B (atypical pancreatitis presentation; median: 1327 mg/L, *p* = 0.0110), and a serum α_1_PI concentration >1579 mg/L (red horizontal line) distinguished both groups of dogs with a sensitivity and specificity of 70% and 100%, respectively. Dashed gray lines indicate the lower limit (750 mg/L) and upper limit (1556 mg/L) of the reference interval for serum α_1_PI concentrations. (**c**) The area under the ROC curve for serum α_1_PI to separate dogs in group A from those in group B was 0.84. The blue line represents the line of identity.

No differences were seen in serum calprotectin and S100A12 concentrations between groups A and B, but dogs in group A (typical presentation of pancreatitis) had significantly higher α_1_PI concentrations (median: 1680 mg/L; range: 1011–2428 mg/L) than dogs in group B (atypical presentation of pancreatitis; median: 1327 mg/L; range: 1208–1543 mg/L, *p* = 0.0110); and a similar trend was seen for serum calprotectin-to-S100A12 ratios (median: 52.4; range: 20.8–84.8 vs. median: 1327 mg/L; range: 1208–1543 mg/L; *p* = 0.0606) (Table 1). As opposed to group A, where 19 dogs (70%) had an increased serum α_1_PI concentration, this marker was not increased in any of the dogs in group B (0%; *p* = 0.0005; Figure 1a). Frequencies of increased serum calprotectin and S100A12 concentrations in group A (for both markers 11/28, 39%) vs. group B (for both markers 4/7, 57%) were not statistically different. A serum α_1_PI concentration >1579 mg/L distinguished dogs with a typical pancreatitis presentation (group A) from those with an atypical presentation of pancreatitis (group B) with a sensitivity of 70% and a specificity of 100% (area under the curve: 84%; Figure 1b–c).

Univariate analysis showed that displaying fewer clinical signs in combination was associated with higher serum calprotectin (*p* = 0.0377) and S100A12 concentrations (*p* = 0.0149) in dogs of both groups combined (Table 3), and this relationship remained significant for the dogs with a typical pancreatitis presentation (group A). However, serum calprotectin and S100A12 concentrations were significantly higher in dogs without vomiting (*p* = 0.0188 and *p* = 0.0133), with a strong similar trend in group A. No such differences were seen for any other clinical signs, the presence of HTG, or a history of receiving a medication known to cause pancreatitis (Table 3). The effect of feeding a diet that is not fat-restricted compared to a low-fat diet could not be evaluated as most dogs were not on a fat-restricted diet. Of the variables with *p* < 0.1 in univariate analysis, only the absence of vomiting remained a significant factor affecting serum calprotectin (*p* = 0.0159) and S100A12 concentrations (*p* = 0.0194) in the multivariate model.

## 4. Discussion

Pancreatitis is a disease with a high mortality rate in dogs, and successful treatment may depend on the early detection of the disease and assessment of disease severity to determine the optimal management plan for an individual patient [39]. Serum cPLI measurement, in combination with the medical history and clinical presentation of the dog and abdominal imaging (i.e., ultrasound), usually provides a reliable diagnosis [2,3,4]. However, consistent with strategies in human medicine, biomarkers that can be routinely determined and help to further classify the disease and distinguish different disease severities would be very useful in small animal clinical practice and could further improve the diagnosis and management of dogs with pancreatitis.

Serum α_1_PI concentrations were found to be increased in about 60% of the dogs in this study, and an increased serum α_1_PI concentration (>1579 mg/L) separated dogs with pancreatitis and an atypical clinical presentation from those with a more typical clinical picture with a specificity of 100% and a sensitivity of 70%. Alpha_1_-PI is a major plasma protease inhibitor [40] and a marker of systemic antiprotease activity, but its role as either a negative or positive acute phase protein in dogs has remained unclear given the limited data available for this biomarker [41,42]. Increased serum α_1_PI concentrations could be explained as reflecting the compensatory protective response to an acute protease-antiprotease imbalance during episodes of pancreatitis, which would support the role of α_1_PI as an acute-phase protein in dogs. In line with this, studies in humans found serum α_1_PI concentrations to increase in proportion to the severity and clinical course of pancreatitis [43,44,45].

This study shows that the systemic inflammatory biomarkers serum calprotectin and S100A12, and α_1_PI as a marker of systemic antiprotease capacity, are commonly increased in Miniature Schnauzers with a diagnosis of pancreatitis. Serum calprotectin and S100A12 appear to be of limited utility as surrogate markers to differentiate presentations of pancreatitis in dogs. However, their relative proportion to each other—expressed as the serum calprotectin-to-S100A12 ratio, which has been shown to be useful to distinguish acute from chronic pulmonary disease [46]—could be more useful given the trend to separate dogs with an atypical pancreatitis presentation from those with a typical clinical presentation. Thus, further longitudinal evaluation of the serum calprotectin-to-S100A12 ratio in larger cohorts of dogs with different presentations and severities of pancreatitis and in comparison to serum CRP concentrations is warranted.

The results of our study are consistent with findings in human pancreatology and intensive care medicine and with the results of recent investigations showing increased serum α_1_PI concentrations in some dogs with acute pancreatitis [42,47]. However, our findings would contrast with the lack of a difference in serum α_1_PI concentrations compared to controls in that study [42] and with the detection of decreased serum α_1_PI concentrations in dogs with non-infectious systemic inflammatory response syndrome (SIRS) or sepsis [41]. More severe episodes of pancreatitis have been associated with significantly larger increases in serum α_1_PI concentrations in people [43,44,45], but an objective assessment of the clinical severity of pancreatitis (i.e., utilization of a validated clinical scoring system) was not included in our investigation, given the lack of a standardized clinical scoring system established to evaluate cases of canine pancreatitis. Previous studies in dogs have attempted to assess pancreatitis severity through histopathologic evaluation and determination of organ failure (e.g., renal and hepatic function), standardized scoring systems using the Ranson or Glasgow scoring systems, extrapolation of the APACHE II (Acute Physiology and Chronic Health Evaluation II) score used in human medicine [48], or including serum C-reactive protein (CRP) concentration as a systemic inflammatory marker in the assessment of pancreatitis severity [12]. The CAPS (Canine Acute Pancreatitis Severity) score and the simplified CAPS (sCAPS) score were developed to predict short-term negative outcomes by assessing four risk factors (presence of SIRS, presence of coagulopathy, increased serum creatinine, and ionized hypocalcemia) [49]. The data obtained in our study warrant further evaluation of serum α_1_PI concentrations and the serum calprotectin-to-S100A12 ratio for their potential diagnostic and prognostic utility in dogs with pancreatitis, particularly when incorporated as additional elements in established clinical scoring systems and when interpreted together with potential risk factors for the development of pancreatitis (e.g., familial or secondary HTG, endocrine disorders, and certain medications).

In conclusion, biomarkers of systemic inflammation and antiprotease activity are commonly increased in Miniature Schnauzers with pancreatitis, which presumably can be extrapolated to other breeds of dogs. Although serum calprotectin and S100A12 concentrations appear to have limited utility in differentiating pancreatitis presentations—at least in Miniature Schnauzers—serum α_1_PI concentrations and potentially also the serum calprotectin-to-S100A12 ratio might present non-invasive surrogate biomarkers to stratify cases of canine pancreatitis (e.g., based on severity). While these markers cannot specifically diagnose pancreatitis as they are not specifically released by pancreatic acinar cells, further studies are needed to determine the clinical utility of these biomarkers in the characterization, monitoring, and prognostication of pancreatitis in dogs.

## Figures and Tables

**Table 1:** Patient characteristics and serum biomarker concentrations for the two groups of dogs included in the study.

Parameter	Typical Clinical Presentation (Group A) ^#^	Atypical Clinical Presentation (Group B)	*p*-Value
***Patient characteristics***			
Age (in years)	8.9 (1.7–12.7)	9.1 (3.3–14.6)	0.4492
Body weight (in kg)	9.2 (6.2–13.3)	8.6 (6.4–14.2)	0.7031
Sex (female/male)	7/11	5/2	0.1394
**No. of clinical signs ^$^**	4 (0–5)	3 (0–5)	0.8194
Vomiting	13/17 (76%)	4/7 (57%)	0.3527
Diarrhea	5/17 (29%)	3/7 (43%)	0.5298
Abdominal pain	10/17 (59%)	2/7 (29%)	0.1726
Anorexia	7/17 (41%)	5/7 (71%)	0.1726
Depression	10/17 (59%)	6/7 (86%)	0.1826
Hypertriglyceridemia ^†^	12/16 (75%)	2/5 (40%)	0.1564
Medications associated with the risk of pancreatitis ^†^	0/15 (0%)	1/6 (17%)	0.1046
Euthanasia	1/18 (6%)	0/7 (0%)	0.412
***Serum biomarkers***			
Serum α_1_PI ^‡^	**1680** (1011–2428) mg/L	**1327** (1208–1543) mg/L	**0.011**
Increased serum α_1_PI ^‡^	**19/27 (70%)**	**0/6 (0%)**	**0.0005**
Serum calprotectin	16.6 (1.2–269.7) mg/L	7.2 (1.3–101.2) mg/L	0.5227
Hypercalprotectinemia	11/28 (39%)	4/7 (57%)	0.3954
Serum S100A12	266 (16 –6464) μg/L	190 (77–2713) μg/L	0.6952
Increased serum S100A12	11/28 (39%)	4/7 (57 %)	0.3954
Serum calprotectin-to-S100A12 ratio	**52.4** (20.8–84.8)	38.1 (16.9–58.9)	**0.0606**

^#^ includes dogs with pancreatitis only (*n* = 24) and dogs with pancreatitis + concurrent condition(s) (*n* = 4); ^$^ available for *n* = 24 dogs (note that only the presence but not the severity of these clinical signs was analyzed); ^†^ available for *n* = 21 dogs; ^‡^ available for *n* = 33 dogs. Values in bold font indicate significant (*p* < 0.05) differences or associations.

**Table 2:** Correlations among serum biomarker concentrations and patient characteristics.

Parameter Correlated with	Serum α_1_PI	Serum Calprotectin	Serum S100A12
Serum α_1_PI		**0.48 (0.0047)**	**0.44 (0.0112)**
Serum calprotectin	**0.48 (0.0047)**		**0.94 (<0.0001)**
Serum S100A12	**0.44 (0.0112)**	**0.94 (<0.0001)**	
Age	−0.26 (0.2291)	0.05 (0.8322)	0.04 (0.8652)
Body weight	−0.24 (0.2784)	−0.31 (0.1363)	−0.39 (0.0586)
No. of clinical signs	−0.20 (0.3745)	**−0.53 (0.0377)**	**−0.49 (0.0149)**

Values in bold font indicate significant (*p* < 0.05) correlations.

**Table 3:** Univariate analyses (*p*-Values) of the effects of different clinical signs on serum biomarker concentrations in dogs with pancreatitis.

Parameter	Serum α_1_PI	Serum Calprotectin	Serum S100A12	Serum Calprotectin-to-S100A12 Ratio
**No. of clinical signs ^$^**	0.3745	**0.0377 ^a^**	**0.0149 ^a^**	0.778
Vomiting	0.6953	**0.0188 ^a^**	**0.0133 ^a^**	0.6566
Diarrhea	0.8112	0.1046	0.1335	0.1046
Abdominal pain	0.4483	0.2366	**0.0885 ^a^**	0.4025
Anorexia	0.3933	0.6236	0.4357	0.665
Lethargy	0.2308	**0.0708 ^a^**	**0.0537 ^a^**	0.7363
Hypertriglyceridemia ^†^	0.3139	0.4334	0.4334	0.351
Medication(s) known to be associated with pancreatitis ^†^	–	0.2155	0.1167	0.8044

^$^ available for *n* = 24 dogs; ^†^ available from *n* = 21 dogs. ^a^ inverse relationship between clinical variables and biomarker levels.

## Data Availability

Data (anonymized for patient and owner data) are available from the second or senior author upon reasonable request.
